# Trial of a family-based education program for heart failure patients in rural Thailand

**DOI:** 10.1186/1471-2261-14-173

**Published:** 2014-12-03

**Authors:** Nittaya Srisuk, Jan Cameron, Chantal F Ski, David R Thompson

**Affiliations:** Centre for the Heart and Mind, Australian Catholic University, Melbourne, VIC Australia; Faculty of Nursing, Suratthani Rajabhat University, Suratthani, Thailand

**Keywords:** Family, Education, Self-care, Health-related quality of life, Heart failure, Thailand

## Abstract

**Background:**

Heart failure (HF) significantly impacts on the daily lives of patients and their carers. In Western society HF education programs have increased patient and carer knowledge and improved health-related quality of life. However, there is a paucity of such evidence in Asia. For example, to date no studies have been conducted in Thailand to investigate the potential benefits of a family-based education program on the health outcomes of HF patients and carers.

**Methods:**

This randomised controlled trial will evaluate the effectiveness of an education program on knowledge, self-care and health-related quality of life of Thai HF patients and their carers. Assessments will be conducted at baseline, three and six months. Participants will be assigned by independent random allocation to an intervention (family-based education plus usual care) or a control (usual care) group. Analyses will be conducted on an intention-to-treat basis.

**Discussion:**

This trial will be the first to evaluate the effectiveness of family-based education for HF patients and carers residing in rural Thailand. It attempts to advance understanding of family-based HF education and address the gap in service provision.

**Trial registration:**

Thai Clinical Trial Registry TCTR20140506003

## Background

Over 23 million people worldwide suffer from heart failure (HF) [1]. Already at epidemic proportions, this significant global public health problem is predicted to escalate exponentially over the next decade [1]. For example, currently in the United States HF affects over five million people [2] and it is expected that by 2030 eight million will be diagnosed with HF [3]. Heart failure is now one of the most common reasons for hospital admissions in older people, resulting in a substantial economic drain on healthcare resources [4].

One way to redress the burden of HF is through implementation of multidisciplinary chronic disease models of care that have been shown to yield significant benefits, compared with usual care, in reducing readmissions and associated costs and improving patient quality of life [5]. Identified as a key component of these programs is patient education specifically directed at promoting self-care behaviours [6]. Most studies investigating HF patient education have been conducted in Europe [7–10], Australia [11–13] and North America [14–17]. This leaves a significant knowledge gap regarding the efficacy of such programs in Asia, for example Thailand.

The Thai Acute Decompensated HEart Failure REgistry (Thai ADHERE) has recognised HF as a major cardiovascular health problem and economic burden [18]. Thai ADHERE epidemiological data were collected from 18 cardiovascular health centres across Thailand. Thai ADHERE found 2,041 HF admissions among 1,612 HF patients during 2006 to 2007. A large proportion of re-hospitalisations were admitted via the emergency department (77%), of which one quarter (25%) required a critical care bed. The absence of HF education programs was identified as a significant factor leading to the high numbers of Thai HF readmissions [18]. Evidence presented from studies conducted in Western countries has demonstrated that many HF readmissions are preventable through patient education targeting self-care [6, 19].

Patient education aims to improve knowledge and skills in order to positively influence attitudes and behaviours, thereby resulting in improved health outcomes [20]. Knowledge can improve an individual’s confidence and sense of control, and with respect to understanding the disease trajectory of HF, can motivate patients to adhere to treatment and lifestyle changes and necessary adaptations [21]. The evidence-base in support of patient education improving HF health outcomes is compelling [5, 6, 22], and has resulted in the inclusion of educational strategies as a key non-pharmacological component within evidenced-based HF practice guidelines [4, 23, 24].

The situation-specific theory of HF self-care provides a framework for understanding and evaluating the competencies of HF patients [25]. Self-care refers to a naturalistic decision-making process involving the choice of behaviours patients adopt in order to maintain physiological stability and responses executed when they occur [26]. Appropriate engagement in HF self-care has the potential to reduce HF readmissions and health care costs, and improve health-related quality of life [27]. In light of this, evidence has also revealed that patients with HF demonstrate difficulties in complying with the recommended self-care regimen [28]. Recently, and importantly, patient knowledge and social support from informal carers are fast being recognised as key to successful self-care [29–32].

The informal carer can be defined as a spouse/partner, family member, friend or neighbour who performs caring without pay, assisting the care recipient with daily activities and/or medicine administration [33]. The role of carers is crucial in HF self-care. A recent systematic review found that the social well-being of HF patients is strongly linked with carers’ support in performing HF self-care [29]. Social support has been found to be a vital resource for patients with HF and used as part of their everyday coping strategies [32]. Patient health outcomes, including health-related quality of life (HRQoL), rehospitalisation, adherence to HF treatment, and optimal engagement in self-care, are strongly associated with the existence of carers [34]. International guidelines now specify that carers be included in the educational processes that promote HF self-care [35–37], though the number of intervention studies that have focused on informal carers of HF are limited [38].

Although the evidence is compelling as to the importance of patient education promoting self-care, most studies investigating patient education have been conducted in Europe, Australia and North America where the characteristics of the population and the culture are often quite different from that in Asia, including Thailand, and especially in rural communities. In Thailand only one published randomised trial of education (coaching by telephone) in HF has been conducted [39], and only two other published studies have explored the notion of education improving knowledge, self-care and HF symptoms [40, 41]. However, limitations of these studies include the use of quasi-experimental designs, small samples, differing theoretical frameworks for developing the educational strategies, and varied settings - home-based to outpatient clinic settings – for conducting them. Moreover, none of these studies included carers, or delivered the education program in the community, nor examined changes in HRQoL in both the patient and carer as an outcome of the education. In light of the incomplete body of knowledge regarding the effectiveness of an education-based intervention for HF patients and carers in Thailand, and the increasing prevalence of HF in Thailand, further research is warranted.

Cultural perspectives become paramount when adapting a Western-based intervention into an Asian country. For example, in Thailand the role of carer is crucial, and the Buddhist concept of karma strongly influences Thai people’s beliefs and their way of thinking and living. Although demographic and socio-economic aspects have changed dramatically in Thailand over recent decades, family members remain the pillar of support for elderly people [42]. Living with older parents, showing respect and taking care of them are considered a normal way of family life and are highly valued in Thailand. Therefore, providing an education program that involves both the patient and carer has the potential to enhance the quality of life for the patient with HF, and for their supporting family [43].

### Aim

The aim of this research is to develop and evaluate a family-based education program for HF patients and their carers residing in rural Thailand.

### Hypothesis

We hypothesise that a family-based education program is effective in improving HF knowledge, self-care behaviour and HRQoL for patients, and perceived control, HF knowledge and HRQoL for their carers living in rural Thailand.

## Methods

### Study design

**Figure 1:**
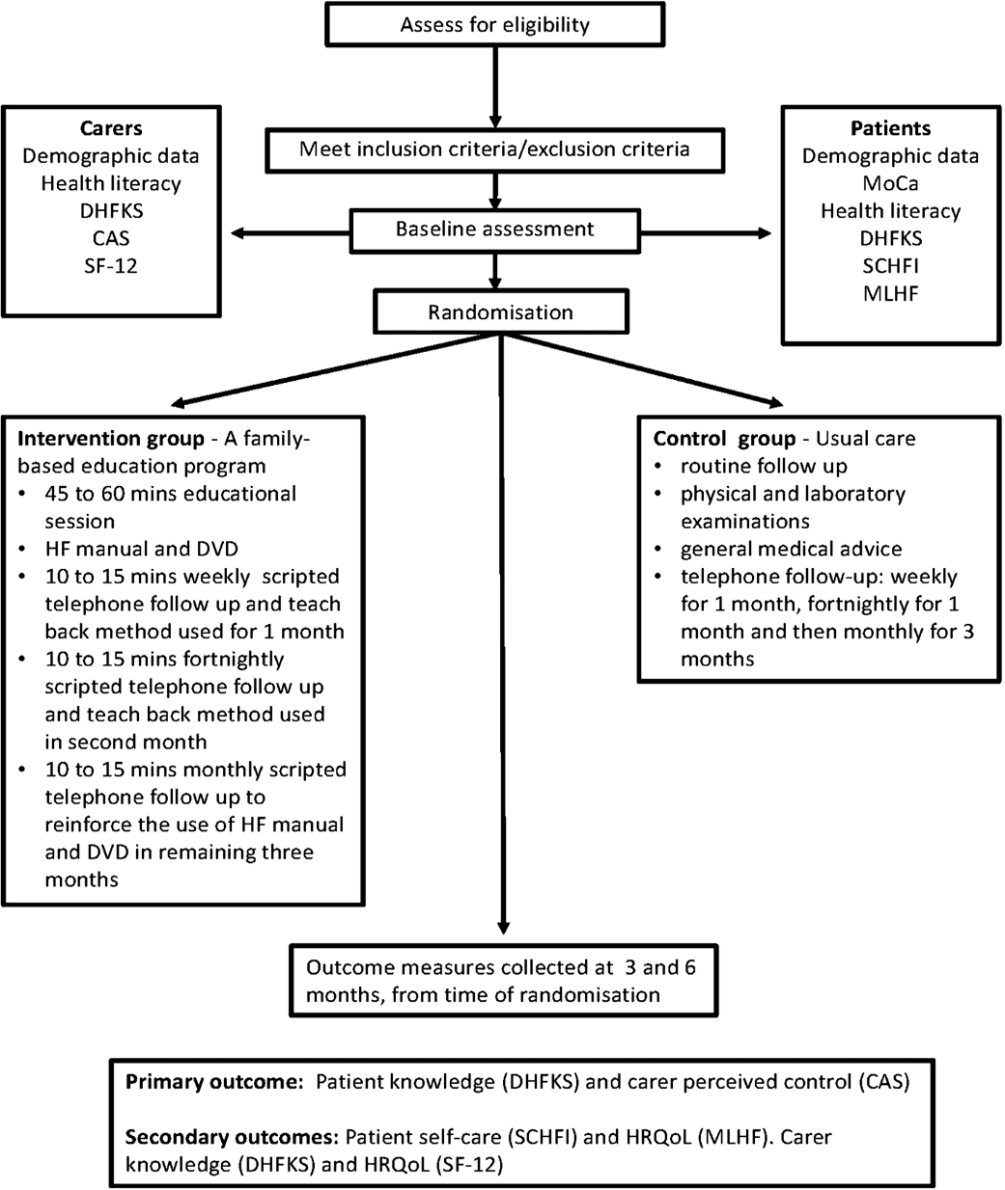
**Study design, flow of participant.**

This is a prospective single-blind randomised controlled trial of family-based education versus usual care for HF patients and their partners residing in rural Thailand. Ethics approval has been obtained from the Human Research Ethics Committees of the Australian Catholic University and Chumphon Hospital and informed consent will be obtained from all participants prior to enrolment. The trial will be conducted in accordance with CONSORT (Consolidated Standards of Reporting Trials) guidelines ([44] See Figure 1).

The setting will be outpatient clinics at one provincial hospital and one community hospital in Chumphon province in the south of Thailand. These clinics were purposively selected on the basis that they are representative of HF patients and care in the rural community. Participants will be assigned by independent random allocation to an intervention (family-based education plus usual care) or a control (usual care) group.

### Participant eligibility

Participants for this study will be a dyad consisting of a patient and their partner or family member that is identified as providing informal care. All participants will need to have sufficient comprehension to read Thai without the need for a translator. The patients will be aged 20 years or over, although with the increasing prevalence of HF in the elderly, it is envisaged that the majority will be above 50 years of age. The inclusion criteria for HF patients are: 1) a primary diagnosis of HF NYHA (New York Heart Association) class I to III confirmed by the treating doctor and determined by a history of typical signs and symptoms and physical examination [45] and, where possible, objective evidence of cardiac dysfunction on echocardiogram; 2) at least one family member residing with them; 3) contactable by telephone at home; and 4) a DVD player at home. The inclusion criteria for carers are: 1) living in the same household as the HF patient or someone of the patient’s own choice; and 2) aged 20 years or over. Participants will be excluded if they reside in an urban area, have a documented history of dementia or severe psychiatric illness, are unable to continue to follow the protocol, and have severe symptoms indicative of acute heart failure [24, 46] or complications from cardiac or disease or other life threatening comorbidities.

### Recruitment

This trial will enroll a minimum of 100 dyads sourced from two rural hospitals in Thailand. Participants will be identified for enrolment by clinical staff based on inclusion criteria. Potential dyads will be approached at their regular outpatient appointment or contacted via telephone and verbally informed of the study. The dyads who agree to participate will receive a verbal and written explanation of the study by the investigator or the attending nurse, allowing them time to consider and freely participate.

### Randomisation

The investigator will derive the random allocation from a computer-generated sequence of random numbers. Each dyad will be randomised at a 1:1 ratio in blocks of ten to either the control (usual care) or intervention (family-based education plus usual care) group. The random allocation will be sealed in an envelope and retained by technical staff at each hospital to give to the research assistants. Once participants have consented to the study and baseline data has been collected, the research assistant will open the sealed envelope indicating the group allocation. In this way, the research assistant collecting baseline data will be unaware of which group participants have been allocated to.

### Intervention

#### Development of the HF education DVD and manual

A HF DVD and accompanying manual was developed by the principal investigator guided by adult learning theory [47] and studies that have investigated the individual learning needs of HF patients, especially in Asia [48–51]. Both the DVD and HF manual have been reviewed by a panel of HF experts in Australia and verified for content and cultural validity by a panel of HF experts in Thailand. The DVD and HF manual have been tested for readability and comprehensibility by three HF patients and carer dyads, who reported both resources to be helpful in gaining knowledge and self-care skills as well as assisting in coping with HF.

The DVD contains nine chapters that explain key aspects in learning to live with and adjust to HF. Chapter headings are: 1) What is HF?; 2) How does HF make you feel?; 3) When you feel sick what should you do?; 4) How you can make your heart feel better?; 5) Your medicine; 6) Your health record; 7) Your HF action plan; 8) Tips for your family and friends; and 9) Conclusion. The DVD was developed in an easy and simple mode for patients and carers to readily absorb the information, using pictures that reflect the Thai cultural context. At the end of each chapter, a reflective question is asked to encourage the patient and their carer to interact and discuss openly potential issues (e.g. which of the symptoms is the hardest for me to manage?).

The written manual is based on the DVD chapters and combines information for both patient and their carers. The manual contains more detail than the DVD, including written material, pictures and health record forms. The HF manual is divided into easily recognisable, colour-coded chapters that correspond to the nine DVD chapters. Both patient and carer will be asked to read each chapter within the manual and also help each other to complete the reflective questions or activities at the end of each section.

#### Intervention group

Treatment fidelity will be used to guide the implementation of the educational program which consists of five processes including: design, training, delivery, receipt, and enactment [52, 53]. The dyads in the intervention will receive their usual care plus a single individualised patient-carer education session. Education sessions will be conducted in the teaching room of the outpatient clinic. Dyads will receive the HF manual and a 45 to 60 minute education session focused on HF self-care. At the end of the session they will receive instructions about using the DVD and manual with their primary carer and/or family members. In addition, the intervention group will receive scripted telephone calls for 15 minutes per week in the first month, fortnightly in the second month, and once a month for the third to sixth month. The purpose of the telephone call is to reinforce, support and counsel each dyad about the HF information provided and assess any ongoing learning needs. The principal investigator will use the teach-back method [54] with each telephone call and give the dyad an opportunity to ask questions. The teach-back method is a technique used by educators to recall, deliver messages, encourage, and check for understanding, with participants asked to repeat the information that has been imparted to them [54]. This technique has been used to assess learning and promote self-care in patients with chronic conditions [54–56]. Specifically, in patients with HF, the teach-back method has been found to be an effective approach used in evaluating and educating patients’ self-care abilities [57, 58].

#### Control group

The control group will receive usual care provided by a clinician that includes: routine follow-up, physical and laboratory examinations, and general medical advice. The carer of the HF patient will receive any additional information if requested. In addition, the control group will receive weekly telephone calls for the first month, fortnightly for the second month, and once a month for the third to sixth month. To reduce the potential of patient contact acting as a confounding variable, the control group will also receive matched telephone calls, although the content of these calls will differ to that of the intervention group by being of a generic nature. Thus the telephone calls for the control group will not contain information based on the family support intervention; instead they will discuss in general terms how the patient and carer are feeling. If the patient’s condition has deteriorated significantly they will be advised to go and see their doctor. In consideration of the principle of fairness, at the end of the six month follow-up period participants in the control group will be offered a copy of the HF DVD and accompanying manual.

#### Data collection

Before randomisation all dyads will complete baseline questionnaires. Outcome measures will be collected at three and six months at the outpatient clinic or via telephone interview. Data will be collected by research assistants who are not aware of study group allocation. Questionnaires will take approximately 45 to 60 minutes to complete.

#### Primary outcome measures

The primary outcome will be HF knowledge as measured by the Dutch Heart Failure Knowledge Scale (DHFKS) in patients. The carers’ primary outcome will be perceived control about managing their family member’s HF as measured by the Control Attitudes Scale (CAS).

#### Secondary outcome measure

Secondary outcomes for patients will be self-care as measured by the Self-Care of HF Index (SCHFI) and HRQoL as measured by the Minnesota Living with HF (MLHF) questionnaire. For carers, secondary outcomes will be HF knowledge as measured by the Dutch Heart Failure Knowledge Scale (DHFKS) and HRQoL as measured by the Short-Form 12-item (SF-12) health survey.

#### Participant descriptive data

Participant clinical and socio-demographic characteristics will also be collected, including: number of social supports; education level; occupational status; health literacy (measured using a single question “How confident are you filling out medical forms by yourself?” [59]); cognitive assessment (measured using the Montreal Cognitive Assessment [60]); comorbid illness burden (measured using Charlson Comorbidity Index [61]); vital signs; cardiac- related history including cardiovascular risk factors and length of time living with HF; New York Heart Association functional classification [62]; and HF current treatment.

### Summary of outcome measure

#### Knowledge

*The Dutch Heart Failure Knowledge Scale (DHFKS)* [63] is a self-report questionnaire consisting of 15 multiple choice items related to: HF in general (4 items), HF treatment (6 items related to diet, fluid restrictions and activity), symptoms and symptom recognition (5 items). The scale has a minimum score of 0 (no correct answer) and a maximum score of 15 (all answers correct), higher scores indicate better knowledge. The tool developed in the Netherlands and tested on 902 HF patients, was found to be reliable (Cronbach’s α .62) and valid [58]. The scale has been shown to be sensitive in differentiating between patients who had and who had not received education and counselling (*p* < .01) and has been widely used in clinical settings to evaluate patients’ HF knowledge [22, 64, 65].

#### Self-care

*The Self-Care of Heart Failure Index (SCHFI)* [66] is an instrument that measures HF self-care behaviours and skills through self-report. The SCHFI comprises 15-items with a four-point Likert response scale. It contains three subscales: self-care maintenance, self-care management, and self-care confidence. Self-care maintenance measures symptom monitoring and compliance with HF treatment in order to prevent worsening symptoms such as checking ankles for swelling and eating a low salt diet. Self-care management measures the capability to recognise changes in HF symptoms, assess the meaning of the changes, and make a judgment on appropriate treatment actions. For example, if a patient experiences weight gain of more than 2 kg in two or three days, an appropriate action would be to take an extra diuretic. Self-care confidence measures perceived control to perform self-care in each phase such as how confident they feel in recognising symptom changes when they occur. Scores from each of the three self-care subscales are transformed to 100-point scales; higher scores reflect better self-care. Self-care management scores are only computed for those patients reporting HF symptoms of ankle swelling or trouble breathing in the previous three months [66]. The SCHFI was selected because it is a reliable measure of self-reported self-care skills and behaviours [66] and has been extensively validated among HF populations around the world. The SCHFI has also been reliably translated into Thai [67]. In the Thai context the tool was administered to 400 HF patients and found to be reliable: Cronbach’s alpha coefficient of 0.85 [67].

#### Health-related quality of life

*The Minnesota Living with Heart Failure (MLHF) questionnaire (MLHF)* [68] is a disease-specific measure of HF HRQoL assessing patients’ perceptions as to the influence of HF on physical, socioeconomic, and psychological aspects of their life. The MLHF consists of 21 questions focused on patients’ perceptions concerning the effects of HF on their physical functioning, such as shortness of breath, fatigue, and peripheral oedema and their emotional life such as memory loss, loss of self-control, and side effects of HF treatment [68]. Patients respond to the 21 items using a 6 -point Likert scale (0 = no; 5 = very much). The total score ranges from 0 to 105; a lower score reflects better HRQoL. Internal consistency reliability of the MLHF using Cronbach’s alpha coefficient was .91 [69]. The Thai version has been used in 422 HF patients, and in pilot testing in a sample of 30, had good reliability with a Cronbach’s alpha coefficient of 0.94 [70].

*The Short-Form 12-item (SF-12) health survey* [71] is a generic measure of HRQoL that will be used in this study as a combination of generic and disease-specific measures has been recommended. The SF-12 [72] is a shortened version of the original SF-36 [73] and consists of 12 items with a 5-point Likert scale (1 = all of the time, 5 = none of the time). The 12 items include the self-assessment of health, physical functioning, physical role limitation, mental role limitation, social functioning, mental health, and pain. The summary score provides an indication of physical and emotional functioning, with higher scores indicating better HRQoL. The Thai version of the SF-12 has been used with 98 HF patients, reliability in this population was 0.83 [74].

#### Perceived control about managing family member’s heart problems

*The Control Attitudes Scale (CAS)* family version will be used only for the carers of patients with HF in this study [75, 76]. The CAS family version consists of eight items scale with a 5-point response scale (1 = strongly disagree, to 5 = strongly agree). The items address how much perceived control or how helpless individuals feel about managing their family member’s heart problems. Higher scores indicate greater perceived control. Internal reliability of the CAS tested in 21 carers of patients with HF was 0.75.

#### Instrument translation

There are three research tools which will be translated into Thai language: the brief screening questions for detecting in adequate health literacy, the DHFKS, and the CAS. The investigator will use the World Health Organisation [77] model process of translation and adaptation of instruments to guide the questionnaire translation.

#### Data analyses and sample size calculation

Data collected will be entered into the Statistical Package for the Social Sciences (SPSS Inc.). Descriptive statistics will be used to analyse demographic data. The sample size calculation is based on changes in HF knowledge. In a previous study investigating HF knowledge as the primary outcome [22]), a sample size of 50 per group and allowing for a 10% attrition rate, had adequate power (0.80) with a two-sided 95% significance to detect a difference of two-points on the DHFKS between the intervention and control conditions in mean post-test versus pre-test changes on the DHFKS and 93% power to detect clinically significant difference in MLHF scores of 6-points. For this study, assuming a medium effect size (0.65) in the between-group differences on the DHFKS and allowing for a 20% attrition rate, the minimal sample size of 40 per group will have sufficient power (0.80). An independent t-test will be used to test the overall differences of the DFHKS, SCHFI, CAS, MLHF and SF-12 within the groups and between the control group and the intervention group at baseline and over three and six months. Two-way repeated measures analysis of variance (ANOVA) will be used to test the difference between groups and change overtime in each group of the main outcomes (DFHKS, SCHFI, CAS, MLHF and SF-12). Treatment failure and withdrawal will be considered on an intention-to-treat basis, with the aim of providing a more realistic estimate of the difference between the two groups.

#### Process evaluation

Ongoing monitoring of program activity in both the intervention and control group will be regularly conducted. Participants will be interviewed when the principal investigator performs a telephone follow-up. Participants in the control group will be asked how satisfied they are with regular telephone contact. The investigator will pilot test the DVD and HF manual in six HF patients and carers prior to implementation of intervention, to ensure their usability and refine if necessary. Participants in the intervention group will be asked how satisfied they are with the DVD and the HF manual and telephone follow-up. At the end of the program participant satisfaction will be measured using a visual analogue scale and participants will be invited to offer additional comments. Completion of activities in the HF manual will be assessed by viewing participants’ HF manual at the six-month assessment. This process assessment is integral to identifying cultural and social facilitators of, and barriers to, the process of lifestyle behaviour change to improve HF risk as a result of this educational intervention.

## Discussion

This trial will test the effectiveness of a family-based education program for HF patients and their carers residing in rural Thailand. Patient education is rapidly becoming recognised as a key component of HF management. Unfortunately, most trials of such programs have been undertaken in Western countries which have considerable differences to Asian countries in terms of, for example, patient characteristics, settings, interventions, and outcome measures. This is the first trial of such a program in rural Thailand taking into account cultural and societal factors.

The trial will not only provide evidence pertaining to the effectiveness of education programs for Thai HF patients, but it will be the first to include family members. We hypothesise that a family-based education program will improve HF knowledge, self-care and HRQoL in patients and carers in rural Thailand. This will be assessed via the implementation of a HF manual and DVD specifically developed to meet the knowledge requirement of HF patients and carers.

## Abbreviations

HF: Heart Failure
Thai ADHERE: Thai Acute Decompensated HEart Failure REgistry
HRQoL: Health-Related Quality of Life
CONSORT: Consolidated Standards of Reporting Trials
NYHA: New York Heart Association
DVD: Digital Video Disc
DHFKS: Dutch Heart Failure Knowledge Scale
CAS: Control Attitudes Scale
SCHFI: Self-Care of HF Index
MLHF: Minnesota Living with Heart Failure questionnaire
SF-12: Short-Form 12-item health survey
ANOVA: ANalysis Of VAriance.
